# Influence of COVID-19 on the Perception of Academic Self-Efficacy, State Anxiety, and Trait Anxiety in College Students

**DOI:** 10.3389/fpsyg.2020.570017

**Published:** 2020-10-09

**Authors:** Inmaculada Alemany-Arrebola, Gloria Rojas-Ruiz, Juan Granda-Vera, Ángel Custodio Mingorance-Estrada

**Affiliations:** ^1^Department of Developmental and Educational Psychology, Universidad de Granada, Granada, Spain; ^2^Department of Didactics and School Organization, Universidad de Granada, Granada, Spain; ^3^Department of Didactics of Musical, Plastic and Body Expression, Universidad de Granada, Granada, Spain

**Keywords:** COVID-19, perceived academic self-efficacy trait anxiety, state anxiety, higher education, health sciences

## Abstract

The worldwide pandemic situation caused by coronavirus disease 2019 (COVID-19) has led to a state of confinement of the population, which has caused, following consulted research, an increase in stress. Faced with this situation, the Spanish university not only had to adapt to the changes derived from the causes of the pandemic but also had to face a new methodological model—e-learning teaching—for which not all teachers and students were prepared. This could cause an increase in stress due to the uncertainty caused by this time of change. This research analyzes the relationship between perceived self-efficacy in the confinement period and the level of trait anxiety (TA) and state anxiety (SA) during COVID-19. Four hundred twenty-seven students from the University of Granada (Spain) participated in this study. The adaptation of two scales that measure perceived academic self-efficacy and anxiety (TA and SA) has been applied. The results indicated that there was an inversely proportional relationship between anxiety and self-efficacy; men showed the highest perception of self-efficacy, while women had higher scores in TA and SA; the latter was accentuated in cases when a relative died. To conclude, students who show a higher level of anxiety (TA and SA) express more negative emotions and also perceive themselves with less academic self-efficacy. Therefore, a stressful situation (pandemic and confinement) together with a critical event (illness and death of a relative/friend due to COVID-19) increases anxiety levels and influences the perception of academic self-efficacy.

## Introduction

March 2020 in Spain, the first period of the state of alarm caused by coronavirus disease 2019 (COVID-19) was declared. This led to the confinement of the population.

Among the containment measures adopted in the university field was the suspension of face-to-face educational activity. As a result, a more online-focused delivery of education was considered. This change affected more than 89.4% of higher education institutions in European countries, Argentina, and Russia (Marinoni et al., 2020). García-Peñalvo (2020) indicated that, although the digitization of the Spanish university began a decade ago, we have focused on face-to-face teaching. However, in this time of isolation, it is necessary to integrate technology into classrooms (Mingorance et al., 2019). But any change generated many uncertainties in the university context. Thus, not attending face-to-face lessons made the American students worried, nervous, and anxious; therefore, 75% of them would like to go back to face-to-face interactions (Kelly, 2020).

Confinement, fear of the unknown, and all these changes caused by the health situation of COVID-19 have increased negative emotions in the Peruvian population, which might harm mental health (Huarcaya-Victoria, 2020). According to Ozamiz et al. (2020), in their research within the Spanish population, at the moment, it is difficult to know the psychological and emotional consequences of this situation, but it is evident that they can lead to anxiety disorders, depression, stress, and increased consumption of harmful substances (Asmundson and Taylor, 2020; Ozamiz et al., 2020; Shigemura et al., 2020; Torales et al., 2020; Valiente et al., 2020; Wang C. et al., 2020).

Other investigations delved into the effects of infectious diseases, such as the research carried out by DiGiovanni et al. (2004) about the severe acute respiratory syndrome (SARS) virus in Toronto, Li et al. (2011) about the H1N1 virus or influenza A in Chinese university students, Taylor et al. (2008) about equine influenza in the Australian population, and Tucci et al. (2017) about the Ebola virus in Occidental Africa and the Zika virus in Brazil and Puerto Rico. They concluded that, during this pandemic situation, the risk of psychological problems increased in the population. Due to the nature of the virus and the fact that is very contagious, among other reasons, the general Chinese population experienced these situations with an increase in anxiety, depression, and other stress reactions (Wang Y. et al., 2020), incrementing psychological anguish if there was a loss of a relative (Mohammed et al., 2015; Ho et al., 2020), results obtained in Chinese and Nigerian populations.

The university environment is not exempt from all these factors. Focusing on the Science, Technology, Engineering and Mathematics (STEM) student body in the United States, these new circumstances originating from COVID-19 was joined to the many demanding aspects of this educational stage, such as academic success (Hall and Sverdlik, 2016). Azad et al. (2017) and the American College Health Association (2019) reported that, faced with the pandemic situation, Turkish and American university students, respectively, had a high risk of suffering anxiety, depression, and panic attacks; these were the most common disorders. In addition, having someone close diagnosed with COVID-19 increased the risk of higher anxiety levels in Chinese population (Cao et al., 2020).

Furthermore, the university context is potentially stressful, and the situation generated by COVID-19 could have a negative impact on students. Although there is little research that analyzes the impact of the pandemic on Chinese students and its influence on academic expectations, some studies concluded that 21.3% of Medicine students showed mild anxiety, 2.7% moderate anxiety, and 0.9% severe levels of anxiety. Having relatives or acquaintances infected with COVID-19 was a risk factor (Cao et al., 2020). In the survey to college students conducted by the American College Health Association (2019), data indicated that 26% of college students reported feeling very depressed compared to 43% who indicated that they were overwhelmed by anxiety. But in addition, the situation of isolation increased psychological pressure (Xiao, 2020); the symptoms persisted even after the confinement (Pfefferbaum and North, 2020).

Regarding the psychological impact, Li et al. (2020) reported that 28.8% of the Chinese respondents suffered anxiety symptoms from moderate to severe and 8.1% responded that they had stress levels (from moderate to severe); Chinese and Canadian women suffered from higher levels of stress, anxiety, and depression (Cao et al., 2020; Taylor et al., 2020). In the same line, Vera-Villaroel (2020) indicated that the stress generated by the global pandemic was related to anxiety, and women presented higher levels than men (Gutiérrez-García and Landeros-Velázquez, 2018). During the COVID-19 crisis, 7% of the general Chinese population suffered symptoms of depression (Kang et al., 2020), increasing anxiety, depression, and outrage (Dubey et al., 2020).

Furthermore, anxiety is indirectly related to academic performance and directly related to the perception of academic self-efficacy (Gutiérrez-García and Landeros-Velázquez, 2018). Therefore, 29% of the Spanish university students with a high level of stress had low levels of self-efficacy (Navarro-Mateu et al., 2020). In this line, anxiety was negatively related to academic self-efficacy (Gutiérrez-García and Landeros-Velázquez, 2018) and positively to state anxiety (SA) in Nigerian university students (Onyeizugbo, 2010).

On the other hand, the perceived academic self-efficacy in the academic environment and the performance of university students are factors that are positively related. This is stated by Colom (2012) and Ahmadi (2020) who found that Iranian students’ self-perceptions about their own ability and competence to perform a certain task is significantly associated with academic performance. A subsequent study confirmed the influence that academic self-efficacy and emotions have on the academic success of university students. Thus, higher levels of positive emotionality were related to higher levels of success (De la Fuente et al., 2019).

At the academic level, academic self-efficacy was related to performance and indirectly influenced academic aspirations (Ahmadi, 2020). In addition, the stress generated by the academic context (overload of tasks, pressure due to work, frequent evaluations, and the pandemic situation, among others) could lower expectations of self-efficacy. Thus, 29% of students with high levels of stress showed low levels of self-efficacy, concluding that there is an inversely proportional relationship (Navarro-Mateu et al., 2020). These findings highlight the importance of these variables for academic performance since those who had great academic self-efficacy also presented more study and cognitive tools that helped organize themselves better (Delgado et al., 2019).

Some research indicated that there were no differences between women and men in academic self-efficacy (Rivera-Heredia et al., 2016; Hernández-Jácquer, 2018); however, Narváez-Olmedo et al. (2018) concluded that it is men who showed the highest perception of academic self-efficacy.

Academic self-efficacy is a variable to be considered in the university context, as it indicates students’ future goals according to their abilities, such as achievement motivation, access to scholarships, academic performance, or permanence in higher education (Borzone, 2017). But, in this time of confinement, when we have quickly moved from face-to-face teaching to remote emergency teaching (Abreu, 2020), it was important to analyze whether this improvised change could affect the expectations of perceived self-efficacy of university students to achieve academic success, since students were not prepared.

There is little research on the impact of the pandemic and its influence on levels of anxiety in university students, and even less related to expectations of academic self-efficacy. For this reason, the objective of this research is to analyze the relationship between perceived self-efficacy and trait anxiety (TA) and SA, during the first moments of COVID-19, since it was an exceptional situation to learn how university students responded to critical situations (related to SA). In addition, the situation of uncertainty and ignorance about the disease was high, generating a great concern in the Spanish population and, more specifically, in university students, given the challenges they faced with the closure of the university.

There were two hypotheses: (1) there is an inversely proportional relationship between academic self-perception and TA and SA; and (2) the students are more sensitive to this situation if they manifest higher TA and also if they or their relatives and friends have suffered from COVID-19.

## Materials and Methods

The design used in this research was a retrospective or *ex post facto* study (Sharma, 2019) with a cross-sectional design to collect data.

### Participants

The research was carried out in the Melilla Campus at the University of Granada (UGR) (Spain). For the selection of the participating sample, a non-probability sampling was used, using the virtual snowball technique (Baltar and Gorjup, 2012).

The selected sample consisted of 427 university students with an average age of 21.30 years (SD = 3.27), ranging 20 years, minimum age is 18 and maximum age is 41. Eighty-four of the participants were men (19.7%), and 342 were women (80.1%). This percentage is a reflection of the feminization of the degrees in nursing and education sciences. A total of 52.3% (*n* = 227) of participants were enrolled in the Faculty of Education and Sport Sciences, 37.6% (*n* = 160) were enrolled in the Faculty of Nursing, 3.3% (*n* = 14) belonged to the Faculty of Social Sciences, and 5.9% (*n* = 25) studied master’s degrees.

In addition, 1.6% (*n* = 7) of the students had suffered/were suffering from COVID-19 compared to 98.4% (*n* = 420) who answered no. In addition, 23.2% (*n* = 99) students affirmed that a friend or a relative had suffered from COVID-19, and of these, 10.8% (*n* = 46) died of it.

### Measures and Procedure

The questionnaires were administered online in March. This was the time of greatest uncertainty and when the state of alarm was declared due to COVID-19. For this purpose, the delegates of the courses from the faculties of the University of Granada at the Melilla Campus (Spain) were contacted *via* e-mail. The questionnaires were uploaded to the Google Form application (free access) with instructions on how to complete it. Furthermore, students were asked to explicitly consent to participate in this experience willingly and anonymously, following the indications given by the Committee on Publication Ethics (COPE).

The questionnaire, https://forms.gle/yP9vgWmAs7e8b6WE7, consisted of three sections:

1. The sociodemographic data section collected information on age, ongoing studies, if he/she suffered/had suffered from COVID-19, and if any relative or friend suffered/had suffered from COVID-19 and if they had died of this cause.
2. The “Adaptation of the Specific Perceived Self-Efficacy Scale in Confinement Situations by COVID-19 (ASPS-COVID-19)” Scale was based on the “Academic Situations Specific Perceived Self-Efficacy Scale” by García-Fernández et al. (2010), consisting of 10 items, with a 4-point Likert-type response format that ranged from 1 (never) to 4 (always). The minimum score was 10 and the maximum was 40, with a reliability of 0.92. The objective was to measure the expectations of self-efficacy in specific situations in the educational context in university students at the time of isolation due to the pandemic. Therefore, the text clarification “during the confinement due to COVID-19” was added to the items of the original scale, keeping the original answer format, so the higher the score, the greater the perceived academic self-efficacy.
3. The “Adaptation of the Trait Anxiety/State Anxiety Scale in Confinement Situations by COVID-19 (ATA/SA-COVID-19)” Scale was based on the Spanish adaptation of the “State-Trait Anxiety Inventory (STAI)” by Spielberger et al. (1982). The scale was composed of 20 items for the Trait Anxiety Subscale (TA) and 20 for the State Anxiety Subscale (SA). The response formats ranged from 0 (hardly ever) to 3 (almost always). The internal consistency indices were 0.96 and 0.88, respectively. The ATA/SA-COVID-19 was made up of 54 items, 28 for TA and 26 for SA. The answer had a 4-point Likert-scale format that ranged from 1 (not at all) to 4 (very often). The minimum score was 28 for TA and 26 for SA. Items were added and repeated in both scales to measure the influence of the pandemic on the emotional and motivational factors that might affect academic goals, such as Anger, Outrage, Boredom, Distraction, Discouragement, and Despondency. Positive items scored inversely (TA: 2, 5, 7, 10, and 15; SA: 1, 2, 5, 8, 10, 11, 15, 16, 20, and 27), so that the higher the score, the higher the anxiety level.

### Data Analysis

Statistical software SPSS 25.0 was used for conducting descriptive analysis of the data, ANOVA test, reliability statistics, and multivariate analysis. Statistical significance was set at *p* < 0.05. Effect size was reported with Cohen’s.

## Results

Table 1 showed the reliability and descriptive analyses of the total score of the different scales used. As it can be observed, the reliability was very high in the three instruments used. In relation to the TA Subscale and in the SA Subscale of the ATA/SA-COVID-19, items 1, 5, 6, 13, and 18 and items 19, 21, 22, and 24 were removed, respectively, as the corrected item–total correlation was under 0.30. The two subscales were composed of 24 items, with a very high inner consistency index, which was measured with the Cronbach’s alpha.

**TABLE 1:** Descriptive statistics for the scales.

Scales		*N*	Mean	Standard deviation	Minimum	Maximum	Skewness	α
ASPS-COVID19 10 items	427	21.11	6.17	10.00	37.00	−0.019	0.914	
ATA/SA-COVID-19	Trait Anxiety (TA) 28 items	427	54.84	11.04	29.00	80.00	−0.080	0.880
	State Anxiety (SA) 22 items	427	74.74	11.37	49.00	102.00	−0.008	0.863

**N*, participants; *α*, Cronbach’s alpha. ASPS-COVID-19, Adaptation of the Specific Perceived Self-Efficacy Scale in Confinement Situations by COVID-19; ATA/SA-COVID-19, Adaptation of the Trait Anxiety/State Anxiety Scale in Confinement Situations by COVID-19.*

In relation to the first hypothesis, the relationship between ASPS-COVID19 and the ATA/SA-COVID-19 subscales was analyzed. The Pearson’s correlation coefficients indicated moderate and significant (*p* < 0.001) relationships between the scales (Table 2), thus increasing anxiety-reduced perceived academic self-efficacy.

**TABLE 2:** Correlation between scales.

Scales		ASPS-COVID-19	ATA-COVID-19	ASA-COVID-19

ASPS-COVID19		1	−0.471	−0.441
ATA/SA-COVID-19	Adaptation of Trait Anxiety (ATA-COVID-19)	−0.471	1	0.774
	Adaptation of State Anxiety (ASA-COVID-19)	−0.441	0.774	1

*ASPS-COVID-19, Adaptation of the Specific Perceived Self-Efficacy Scale in Confinement Situations by COVID-19; ATA/SA-COVID-19, Adaptation of the Trait Anxiety/State Anxiety Scale in Confinement Situations by COVID-19.*

Furthermore, in relation to the ASPS-COVID-19 scale, students were classified into three groups: low perception of academic self-efficacy (means below the 20th percentile, $\bar{x}$ ≤ 16), moderate level of self-efficacy (means between the 21st percentile to the 79th percentile, $\bar{x}$ ≥ 17.00 to $\bar{x}$ ≤ 26.00), and high level of academic self-efficacy ($\bar{x}$ ≥ 27.00). The data showed that the students with the lowest levels of self-efficacy were 99 (23.2%), of which 42 (42.4%) presented very high levels of both TA and SA; 39 women and three men presented the highest level of SA. In addition, one student had suffered from the disease, 19 had a relative who had suffered from it, of whom five had died from COVID-19.

The inferential analysis based on the sex variable showed significant differences. Women showed lower levels of self-efficacy than men (${\bar{x}}_{\text{MEN}}$ = 14.73; ${\bar{x}}_{\text{WOMEN}}$ = 12.91; *t* = 3,295; *p* < 0.005;_dCOHEN_ = 0.717; r_SIZEEFFECT_ = 0.337). The size effect was large. Regarding whether any relative had died, data indicated that the students who had lost a relative scored lower in the perception of academic self-efficacy (x_YES DECEASED_ = 11.69; x_NODECEASED_ = 13.52; *t* = −2.243; *p* < 0.05; d_COHEN_ = −0.708; r_SIZEEFFECT_ = 0.334). The size effect was large.

Multivariate inferential analyses, using SA and TA as covariates and their influence on perceived self-efficacy, showed significant differences (*F*_2,426_ = 7.57, *p* < 0.000; ηp2parcial = 0.035), which indicated that the higher the levels of TA and SA, the worse the perception of self-efficacy in academic performance.

Regarding the second hypothesis, firstly, in the TA and SA subscales, students were classified into three groups in such a way that the first group, with a low level of anxiety, was the one that obtained average scores below the 20th percentile. The group with moderate anxiety level ranged from the 21st to the 79th percentile. Last, the third group, with high anxiety levels, obtained means over the 80th percentile. These were the criteria for the three scales. In Table 3, students are presented according to their level of TA and SA.

**TABLE 3:** Description of the students according to their level of TA and SA (*N* = 427).

		Trait Anxiety (TA)			Total
		Low levels (PC < 20) $\bar{x}$ ≤ 51.00	Medium levels PC < 21 a PC > 79 $\bar{x}$ ≥ 52; $\bar{x}$ ≤ 75.00	High levels PC > 80 $\bar{x}$ ≥ 76.00	
State anxiety (SA)	Low levels (PC < 20) $\bar{x}$ ≤ 57.00	*N* = 80	*N* = 26	*N* = 3	*N* = 109
		18.7%	6.1%	0.7%	25.5%
	Medium levels PC < 21 a PC > 79 $\bar{x}$ ≥ 58.00; $\bar{x}$ ≤ 76.00	*N* = 29	*N* = 144	*N* = 33	*N* = 109
		6.8%	33.7%	7.7%	48.2%
	High levels PC > 80 $\bar{x}$ ≥ 77.00	*N* = 0	*N* = 22	*N* = 90	*N* = 109
		0.0%	5.2%	21.1%	26.2%
Total	109	192	126	427	
	25.5%	45.0%	29.5%	100%	

*PC, percentile; *N*, participants; $\bar{x}$, mean.*

In addition, the students who presented high scores on the TA Scale were selected (PC80 $\bar{x}$ ≥ 76). They were a total of 89 (21.07% of the total sample). In this group, nine were men (10.1%) and 80 were women (89.9%), only two university students (one man and one woman) had COVID19 (2.2%). Furthermore, 39 students had a relative or a friend who had suffered from the disease (43.8%), of whom 11 died (12.4%). Of the 89 university students, 79 (88.8%) also showed high levels of SA (PC80 $\bar{x}$ ≥ 77) and 10 (14.6%) moderate levels. Sixty-seven were women (83.8%) and nine were men (16.2%); two of them had suffered from the disease. In relation to whether a relative/friend suffered/had suffered from COVID-19, 35 answered yes and 12 reported they died of the virus.

In the analyses of the students (*n* = 427), TA and SA showed that the SA was high in this pandemic time during the confinement, the analyzed data showed significant differences, increasing the SA in this pandemic time (TA = 63.74; SA = 66.55; *t* = −7.212; *p* < 0.005; d_COHEN_ = −0.206; r_SIZEEFFECT_ = −0.102); the size effect was medium. Although there were no significant differences, the SA was higher in the students who had a sick relative who died of COVID-19 (TA = 63.06; SA = 67.46; *t* = −1.101; *p* > 0.05). The results of the students with high levels of SA (*n* = 109) indicated that a 100% felt worried and nervous, 90.8% felt distressed, and 80% felt helpless in the situation.

Among the characteristics presented by the students with high levels of TA (*n* = 126) and in relation to their teaching-learning process, the data indicated that they were unfocused (91.1%), unmotivated (98.8%), and discouraged (100%) in the situation they were experiencing (95%). In relation to how they experienced the confinement, 91% felt angry, 85% outraged, and 80% felt unhappy about the pandemic.

## Discussion and Conclusion

The objective of this research is to analyze the relationship between perceived self-efficacy and TA and SA during the first moments of COVID-19. With this objective, the “Academic Situations Specific Perceived Self-Efficacy Scale” and “State-Trait Anxiety Inventory (STAI)” questionnaires were adapted.

The reliability found in the ASPS-COVID-19 was similar to that obtained by García-Fernández et al. (2010). In the same way, the ATA/SA-COVID-19 Subscales obtained high internal consistency indices similar to Fonseca-Pedrero et al. (2012).

Regarding the first hypothesis, the data showed that there was an inversely proportional relationship between the perception of academic self-efficacy and anxiety, both TA and SA, results in line with the research of Gutiérrez-García and Landeros-Velázquez (2018), Navarro-Mateu et al. (2020), and Colom (2012). One explanation might be that anxiety is related to both cognitive and motivational and emotional processes. In this way, negative emotions such as tension about how students experienced the pandemic and a high level of concern could decrease the levels of achievement and self-efficacy (De la Fuente et al., 2019). But also, university students with high levels of anxiety showed high scores in demotivation, discouragement, and boredom, results in line with the research of Vancouver (2018). These motivational characteristics could negatively affect the expectations of self-efficacy that, together with the demands of the university context, could interfere with their academic aspirations (Ahmadi, 2020). In addition, the psychological pressure of the confinement (Xiao, 2020) was joined to the academic demands, workload, and online evaluations that could negatively affect the perception of academic self-efficacy (Navarro-Mateu et al., 2020), generating higher levels of anxiety in university students (Huarcaya-Victoria, 2020). In addition, not attending classes due to the closure of universities was a factor of concern and anxiety (Kelly, 2020), since the work methodology changed (Abreu, 2020) when the students were not prepared.

As for the second hypothesis, there was a directly proportional relationship between TA and SA. The students with the highest TA also increased their level of SA during COVID-19. This might be due to the fact that the pandemic generated a stressful situation causing highly negative emotions, mainly in students who had someone close diagnosed with COVID-19 (Cao et al., 2020) and those who reported the death of a relative or a friend (Mohammed et al., 2015; Ho et al., 2020). Thus, the data showed that students with high levels of SA indicated feeling distressed and helpless in this situation, as reported by the American College Health Association (2019).

Regarding the analyses of the sex variable, men showed a better perception of self-efficacy, data consistent with those found by Narváez-Olmedo et al. (2018). On the other hand, women presented higher levels of anxiety, as reflected by Gutiérrez-García and Landeros-Velázquez (2018), Cao et al. (2020), and Taylor et al. (2020). Their SA increased in stressful situations, such as the death of a relative (Ho et al., 2020), which directly affected the perception of self-efficacy; this result coincided with Gutiérrez-García and Landeros-Velázquez (2018) and De la Fuente et al. (2019) who came to the conclusion of the relationship between self-efficacy and emotionality. Gender differences in academic self-efficacy could be associated with socialization processes and the perpetuation of gender stereotypes in young university students, learned within a sociocultural context.

Some limitations must be acknowledged: first, the type of sampling, but given the situation of confinement, it was the only possibility to obtain data. These data were collected at the beginning of the pandemic. The cross-sectional methodology of this study was necessary and mandatory, but it has been compared with other investigations. For future studies, stratified non-probability sampling will be used and the number of participants will be increased. Secondly, SA and TA and their relationship with the perception of academic self-efficacy have been analyzed, but it is still necessary to include other complementary instruments, such as effects of changes in learning through academic performance (grades), resilience, self-concept, and cognitive-motivational factors that might affect the variables studied. It will be necessary to continue working on the adaptation of the instrument used as well. Third, we did not analyze the effect of the methodological change and how it affects TA and SA and the perception of academic self-efficacy in the e-learning model, so it should be studied in subsequent investigations.

To conclude, students who show a higher level of anxiety (TA and SA) express more negative emotions and also perceive themselves with less academic self-efficacy. Therefore, a stressful situation (pandemic and confinement) together with a critical event (illness and death of a relative/friend due to COVID-19) increases anxiety levels and influences the perception of academic self-efficacy.

Thus, students in exceptional situations increase their levels of anxiety and reduce their perception of academic self-efficacy that can put their permanence in higher education at risk. Therefore, it is necessary to follow up on university students after confinement in order to explore the causes of anxiety and the influence that COVID-19 had on their academic performance and on their permanence in university.

## Data Availability Statement

The raw data supporting the conclusions of this article will be made available by the authors, without undue reservation.

## Ethics Statement

Ethical review and approval was not required for the study on human participants in accordance with the local legislation and institutional requirements. Written informed consent for participation was not required for this study in accordance with the national legislation and the institutional requirements.

## Author Contributions

IA-A contributed to the research conception and design, data analysis and interpretation, statistical analysis, and critical revision of the manuscript for important intellectual content. GR-R contributed to obtaining information, reading the manuscript, and critical revision of the manuscript for important intellectual content. JG-V contributed to obtaining information, reading the manuscript, and critical revision of the manuscript for important intellectual content. ÁM-E contributed to the research conception and design, obtaining information, data interpretation, reading the manuscript, and critical revision of the manuscript for important intellectual content. All authors contributed to the article and approved the submitted version.

## Conflict of Interest

The authors declare that the research was conducted in the absence of any commercial or financial relationships that could be construed as a potential conflict of interest.
