# Supplementary material for: Influence of COVID-19 on the Perception of Academic Self-Efficacy, State Anxiety, and Trait Anxiety in College Students
Source: Front Psychol. 2020 Oct 9;11:570017. doi: 10.3389/fpsyg.2020.570017 (PMC7586314; doi:10.3389/fpsyg.2020.570017)
Supplement: Supplementary file 1 [file Data_Sheet_1.docx]

**Supplementary Material**

Supplement to: Alemany et al. “Influence of COVID-19 on the perception of academic self-efficacy, state anxiety and trait anxiety in college students"

**Table of contents**

**MATERIALS AND METHOD**

**Participants**

**TABLE 1:** Study participants according to the faculty, sex and academic year variables.

**TABLE 2:** Study participants according to whether they suffered from COVID-19 in relation to the sex variable.

**TABLE** **3:** Study participants that had a relative/friend who suffered from COVID-19 and died of it.

**Measures and Procedure**

**Appendix A:** The questionnaire

**MATERIALS AND METHOD**

**Participants**

| **TABLE 1** Study participants according to the faculty, sex and academic year variables | | | | | | | |
| --- | --- | --- | --- | --- | --- | --- | --- |
| **Sex** | **Academic Year** | **N** | **Faculty** | | | | Total |
|  |  |  | **Education and Sport Sciences** | **Health** | **Social Sciences** | Master |  |
| MAN | 1st | N | 18 | 10 | 1 | 3 | 32 |
|  |  | % | 21.4% | 11.9% | 0.8% | 3.6% | 36.9% |
|  | 2nd | N | 10 | 6 | 1 | 3 | 20 |
|  |  | % | 11.9% | 7.1% | 1.2% | 3.6% | 23.8% |
|  | 3rd | N | 8 | 4 | 0 | 0 | 12 |
|  |  | % | 9.5% | 4.8% | 0.0% | 0,0% | 14.3% |
|  | 4th | N | 13 | 6 | 0 | 1 | 20 |
|  |  | % | 15.5% | 7.1% | 0.0% | 1.2% | 23.8% |
| Total | | N | 49 | 26 | 2 | 7 | 84 |
|  |  | % | 58.3% | 31.0% | 2.4% | 8.3% | 100.0% |
| WOMAN | 1st | N | 75 | 50 | 3 | 16 | 144 |
|  |  | % | 21.9% | 14.6% | 0.9% | 4.7% | 42.1% |
|  | 2nd | N | 42 | 36 | 0 | 0 | 78 |
|  |  | % | 12.3% | 10.5% | 0.0% | 0.0% | 22.8% |
|  | 3rd | N | 38 | 23 | 2 | 0 | 63 |
|  |  | % | 11.1% | 6.7% | 0.6% | 0.0% | 18.4% |
|  | 4th | N | 23 | 25 | 7 | 2 | 57 |
|  |  | % | 6.7% | 7.3% | 1.8% | 0.6% | 16.4% |
| Total | | N | 178 | 134 | 12 | 18 | 342 |
|  |  | % | 52.0% | 39.2% | 3.5% | 5.3% | 100.0% |
| **Total** | | N | 227 | 160 | 14 | 25 | 426 |
|  |  | % | 53.3% | 37.6% | 3.3% | 5.9% | 100.0% |

| **TABLE 2** Study participants according to whether they suffered from COVID-19 in relation to the sex variable | | | | | |
| --- | --- | --- | --- | --- | --- |
|  | | | **SEX** | | **Total** |
|  |  |  | **MAN** | **WOMAN** |  |
| **Suffered or had suffered from COVID-19** | **YES** | N | 5 | 2 | 7 |
|  |  | % | 1.2% | 0.5% | 1.6% |
|  | **NO** | N | 79 | 341 | 420 |
|  |  | % | 18.5% | 79.9% | 98.4% |
| **Total** | | N | 84 | 343 | 427 |
|  |  | % | 19.7% | 80.3% | 100.0% |

| **TABLE** **3** Study participants that had a relative/friend who suffered from COVID-19 and died of it | | | | | |
| --- | --- | --- | --- | --- | --- |
|  | | | **Relative/Friend suffers from**  **COVID-19** | | **Total** |
|  |  |  | **SÍ** | **NO** |  |
| **Relative/Friend died of**  **COVID-19** | **YES** | N | 32 | 14 | 46 |
|  |  | % | 7.5% | 3.3% | 10.8% |
|  | **NO** | N | 67 | 314 | 381 |
|  |  | % | 15.7% | 73.5% | 89.2% |
| **Total** | | N | 99 | 328 | 427 |
|  |  | % | 23.2% | 76.8% | 100.0% |

**Appendix A**

**Measures and Procedure**

The questionnaire, <https://forms.gle/yP9vgWmAs7e8b6WE7>, consisted of three sections:

1. The Sociodemographic data section collected information on age, ongoing studies, if he/she suffered / had suffered from COVID-19, if any relative or friend suffered / had suffered from COVID-19 and if they had died of it.
2. The "Adaptation of the Specific Perceived Self-efficacy Scale in Confinement Situations by COVID-19 (ASPS-COVID-19)" scale was based on the "Academic Situations Specific Perceived Self-efficacy Scale" by García-Fernández et al. (2010) consisting of 10 items, with a 4-point Likert-type response format that ranged from one (never) to four (always). The minimum score was 10 and the maximum was 40, with a reliability of 0.92. The objective was to measure the expectations of self-efficacy in specific situations of the educational context in university students at the time of isolation due to the pandemic. Therefore, the text “during the confinement due to COVID-19” was added to the items of the original scale, keeping the original answer format, so the higher the score, the greater the perceived academic self-efficacy.
3. The "Adaptation of the State Anxiety / Trait Anxiety Scale in Confinement Situations by COVID-19 (ATA/SA-COVID-19)" scale was based in college students of Fonseca-Pedrero et al (2012) of the "State-Trait Anxiety Inventory (STAI)" by Spielberger et al. (1982). This instrument measures two independent constructs of anxiety: SA, transient, and TA, relatively stable (Fonseca-Pedrero et al., 2012). The original Scale was composed of 20 items for the Trait Anxiety Subscale (TA) and 20 for the State Anxiety Subscale (SA). The answer formats ranged from 0 (hardly ever) to 3 (almost always). The total score could range from 0 (not anxious at all) to 60 (maximum level of anxiety). The internal consistency indices were 0.96 and 0.88, respectively. The ASA/TA-COVID-19 was made up of 54 items, 28 for TA and 26 for SA. The answer had a 4-point Likert-scale format that ranged from 1 (not at all) to 4 (very often). The minimum score was 28 for TA and 26 for SA. Items were added and repeated in both scales to measure the influence of the pandemic on emotional and motivational factors that might affect academic goals, such as: Anger, Outrage, Boredom, Distraction, Discouragement, and Despondency. Positive items scored inversely (TA: 2, 5, 7, 10 y 15 y SA: 1, 2, 5, 8,10, 11, 15, 16, 20 y 27), so that the higher the score, the higher the anxiety level.

For the analysis of the data, the statistical analysis software SPSS (Statistical Package for the Social Sciences) in version 25.0 was used, descriptive statistics and reliability analyses were performed. The adjustment of the normal distribution of the scores was verified using the Kolmogorov-Smirnov test with the Lilliefors and Shapiro-Wilk correction; regarding the homoscedasticity, the statistical analyses of contrasts were performed using the Levene statistic (t-Student, ANOVA and multivariate analysis). To detect significance, a 95% confidence interval was used.
