# Supplementary material for: Influence of COVID-19 on the Perception of Academic Self-Efficacy, State Anxiety, and Trait Anxiety in College Students
Source: Front Psychol. 2020 Oct 9;11:570017. doi: 10.3389/fpsyg.2020.570017 (PMC7586314; doi:10.3389/fpsyg.2020.570017)
Supplement: Supplementary file 2 [file Data_Sheet_2.docx]

**Highlights**

Alemany et al. “Influence of COVID-19 on the perception of academic self-efficacy, status anxiety and trait anxiety in college students"

- Higher levels of TA and SA were associated with a worse self-efficacy

perception

- There is a directly proportional relationship between TA and SA.
- Students with the highest TA during COVID-19 increased their level of SA
- Men perceived themselves more competent, increasing as they advance in the academic years
- The illness and death of a family member/friend from COVID-19 increased anxiety levels
